# Pangenome insights into structural variation and functional diversification of barley CCT motif genes

**DOI:** 10.1002/tpg2.70098

**Published:** 2025-08-26

**Authors:** Zihao Zhu, Nils Stein

**Affiliations:** ^1^ Leibniz Institute of Plant Genetics and Crop Plant Research (IPK) Seeland Germany; ^2^ Crop Plant Genetics, Institute of Agricultural and Nutritional Sciences Martin‐Luther‐University of Halle‐Wittenberg Halle (Saale) Germany

## Abstract

CONSTANS, CONSTANS‐LIKE, TIMING OF CAB EXPRESSION1 (CCT) motif genes play a key role in barley (*Hordeum vulgare* L.) development and flowering, yet their genetic diversity remains underexplored. Leveraging a barley pangenome (76 genotypes) and pan‐transcriptome (subset of 20 genotypes), we examined CCT gene variation and evolutionary dynamics. Motif‐based searches, combined with genome assembly validation, revealed annotation limitations and novel frameshift variants (e.g., *HvCO10*, where *Hv* is *Hordeum vulgare* L.), indicating active diversification. Pangenome‐wide phylogenetic analysis identified clade‐specific domain expansions, including B‐box domain additions in *HvCO* clades. Tissue‐specific expression patterns further supported functional divergence among paralogs. Notably, *VRN2*, a canonical floral repressor associated with winter growth, was retained in spring genotypes, challenging its presumed exclusive role in vernalization. Discrepancies between *VRN1* expression, *VRN2* deletion, and growth habit implicated additional regulatory mechanisms. These findings highlight the power of pangenomes in resolving gene family complexity, refining annotations, and advancing the understanding of CCT genes to enhance barley resilience and adaptability.

## Core Ideas

- Gene family studies require genomic and transcriptomic integration to ensure accurate annotations.
- Barley pangenome reveals variation and diversification dynamics in CONSTANS, CONSTANS‐LIKE, TIMING OF CAB EXPRESSION1 (CCT) motif genes.
- *VRN2* presence in spring barley challenges its presumed role in growth habit determination.
- *VRN1* expression‐growth habit mismatches point to uncharacterized vernalization pathways in barley.

**Abbreviations**
BAC: bacterial artificial chromosome
BLAST: basic local alignment search tool
BPGv2: barley pangenome version 2
CCT: CONSTANS, CONSTANS‐LIKE, TIMING OF CAB EXPRESSION1
CMF: CCT motif family
CNV: copy number variation
CO: CONSTANS
COL: CONSTANS‐LIKE
FT: FLOWERING LOCUS T
*Hv*
: *Hordeum vulgare* L.
indel: insertion/deletion
PAV: presence/absence variation
PCA: principal component analysis
Ppd‐H1: photoperiod‐H1
PRR: pseudo‐response regulator
RTD: reference transcript dataset
TOC1: TIMING OF CAB EXPRESSION1
ZCCT: zinc‐finger CCT motif protein
ZT: Zeitgeber time

## 1 INTRODUCTION

The CONSTANS (CO), CONSTANS‐LIKE (COL), and TIMING OF CAB EXPRESSION1 (TOC1) or CCT motif genes are a highly conserved family of plant genes that regulate critical developmental and physiological processes, including flowering time, circadian rhythms, photoperiodic responses, and abiotic stress tolerance (Cockram et al., 2007; Putterill et al., 1995). These genes are characterized by a conserved CCT domain located at their C‐terminus, which plays a role in DNA binding and protein–protein interactions (Shen et al., 2020). The CCT family is classified into three major clades based on their N‐terminal domains: the CCT motif family (CMF), COL, and pseudo‐response regulator (PRR). COL proteins, such as CO in Arabidopsis (*Arabidopsis thaliana* (L.) Heynh.) and Heading date 1 (Hd1) in rice (*Oryza sativa* L.), contain one or two B‐box‐type zinc finger domains (Putterill et al., 1995; Yano et al., 2000). CMF proteins, including grain number, plant height, and Heading date 7 (Ghd 7) in rice (Weng et al., 2014), lack a conserved N‐terminal domain. PRR proteins, such as TOC1/PRR1 in Arabidopsis and Ghd7.1 (OsPRR37) in rice, contain pseudo‐receiver domains (Murakami et al., 2005; Strayer et al., 2000). CCT genes, distinguished by their evolutionarily conserved sequences and diverse functions, represent prime targets for improving yield and resilience in cereals (reviewed in H. Liu et al., 2020).

Recent studies in barley (*Hordeum vulgare* L.), a widely cultivated cereal crop, have revealed the role of CCT motif genes in regulating important agronomic traits. For example, *HvCMF4* (where *Hv* is *Hordeum vulgare* L.) is essential for floral development, and mutations in this gene cause primordia abortion and pollination failure by impairing rachis greening and plastidial energy supply (Huang et al., 2023). Other *CMF* genes, such as *HvCMF3* and *HvCMF7*, are crucial for chloroplast development with *HvCMF7* being important for chloroplast ribosome biogenesis and *HvCMF3* regulating chloroplast formation (M. Li et al., 2019, 2021). Mutations in *HvCMF7* result in variegated leaves, while defects in *HvCMF3* lead to chlorosis and thylakoid abnormalities, highlighting their distinct but overlapping functions. *HvCO1* and *HvCO2*, barley's closest homologs of *AtCO*, regulate flowering by activating *HvFT1* (where FT is FLOWERING LOCUS T) in response to photoperiod (Campoli, Drosse, et al., 2012). *HvCO1* accelerates flowering under both long and short days, while *HvCO2* promotes flowering in a *Ppd‐H1* (a PRR domain gene)‐dependent manner (where Ppd‐H1 is photoperiod‐H1) (Campoli, Shtaya, et al., 2012; Turner et al., 2005). In winter barley, *HvFT1* activation is controlled by vernalization. Prior to vernalization, VERNALIZATION 2 (VRN2), a zinc‐finger CCT motif protein (ZCCT), acts as a floral repressor, preventing premature flowering (Mulki & von Korff, 2016; Yan, Loukoianov, et al., 2004). After vernalization, *VRN1*, a MADS‐box transcription factor, suppresses *VRN2* and activates *HvFT1*, promoting flowering through a positive feedback loop (C. Li et al., 2015; Yan et al., 2006). Phylogenetic and comparative analysis of CCT motif genes across species suggests they originated before the monocot/dicot divergence (Cockram et al., 2012). However, many *CMF* and *CO* genes within these families still have undefined functions, and the genetic diversity of these genes, especially in relation to global barley diversity, has yet to be fully explored.

Genome‐wide characterization of gene families is a fundamental approach to understanding gene functions and their evolutionary dynamics. Early research, including the first characterization of barley *COL* genes, primarily relied on bacterial artificial chromosome (BAC) libraries (Faure et al., 2007; Griffiths et al., 2003; Yan, Loukoianov, et al., 2004; Yu et al., 2000). With the increasing availability of complete genome sequences, these early methods have been greatly improved. For instance, the spring barley cultivar Morex was the first barley genome to be fully sequenced (Mascher et al., 2017) and subsequently better annotated (Monat et al., 2019). While these genomic resources enabled the development of a comprehensive barley gene family database (T. Li et al., 2022), the reliance on short‐read sequencing for genome assembly has posed challenges in accurately resolving complex genomic regions (reviewed in Michael & VanBuren, 2020), such as the structural variations detected subsequently (Jayakodi et al., 2020). Furthermore, single‐genotype‐based analyses are limited by the absence of key genes in the reference genotype. For example, *VRN2*, an essential regulator of vernalization and flowering time in winter barley, is absent from the Morex BAC library (Yan, Loukoianov, et al., 2004). These limitations have hindered the full characterization of gene families and their functional diversity.

The recent development of a barley pangenome, which includes 76 diverse genotypes assembled using long‐read high‐fidelity sequencing, offers a transformative resource for genomic research (Jayakodi et al., 2024). This high‐quality pangenome not only provides improved genome assemblies but also offers detailed gene annotations and orthologous relationships across genotypes. The pangenome incorporates genotypes with diverse phenotypic traits, such as growth habit (winter or spring), presenting a unique opportunity to explore the genetic basis of these traits. However, the link between these phenotypic differences and genetic variations in CCT motif genes remains unclear, despite their known roles in plant architecture, flowering time, and vernalization regulation.

In this study, we adopt a pangenome perspective to classify CCT motif genes in barley, aiming to elucidate their genetic diversity, evolutionary patterns, and functional roles across diverse genotypes. By moving beyond traditional protein database‐based analysis, we validated presence/absence variations (PAVs) and copy number variations (CNVs) directly from genome assemblies. This approach revealed significant genetic features, including potential misannotations in *HvCO1*, natural frameshift mutations in *HvCO10*, and CNVs in *VRN2*. Notably, complete copies of *VRN2* were identified in multiple spring barley genotypes, challenging the long‐standing assumption that the absence of *VRN2* is the sole genetic determinant of vernalization requirement.

## 2 MATERIALS AND METHODS

### 2.1 Identification of CCT motif genes in the barley pangenome

All proteins containing CCT (Pfam ID: PF06203), B‐box (PF00643), and PRR (PF00072) domains were identified using HMMER/3.1b2 (Finn et al., 2011) based on the barley pangenome version 2 (BPGv2) protein sequence database (panbarlex.ipk‐gatersleben.de; Jayakodi et al., 2024). Overlapping domain sets were used to classify candidates into HvCMFs (CCT only), HvCOs (CCT with B‐box), and HvPRRs (CCT with PRR) (Supporting Information S1). Proteins with potential CCT domain deletions but retaining B‐box or PRR domains were also retrieved from the corresponding HMMER results. To validate HMMER‐based classification, InterProScan/5.57 (Jones et al., 2014) was conducted using the MorexV3 protein database (Jayakodi et al., 2024), targeting CCT (IPR010402), B‐box (IPR000315), and PRR (IPR017053) domains. A few candidates, including functionally annotated *TIFY* genes and *FAR1‐related sequence 5*, were initially identified as containing potential CCT domains but were excluded due to high *E*‐values (>1).

**Core Ideas**

- Gene family studies require genomic and transcriptomic integration to ensure accurate annotations.
- Barley pangenome reveals variation and diversification dynamics in CONSTANS, CONSTANS‐LIKE, TIMING OF CAB EXPRESSION1 (CCT) motif genes.
- *VRN2* presence in spring barley challenges its presumed role in growth habit determination.
- *VRN1* expression‐growth habit mismatches point to uncharacterized vernalization pathways in barley.

Gene nomenclature was first assigned to candidates identified in winter barley cultivar Akashinriki using BLASTp/2.13.0 (where BLASTp is basic local alignment search tool, protein) (Camacho et al., 2009) against published sequences (Campoli, Drosse, et al., 2012; Cockram et al., 2012; Griffiths et al., 2003). Since the phylogenetic relationships of barley GATA candidates were unclear compared to Arabidopsis and rice (Reyes et al., 2004), they were sequentially named as HvGATA1 (orthogroup ID: N0.HOG0052967), HvGATA2 (N0.HOG004833), HvGATA3 (N0.HOG0057350), and HvGATA4 (N0.HOG0060472). These names were then assigned to corresponding candidates in the other 75 genotypes based on orthologous relationships (Jayakodi et al., 2024). At this stage, the candidates remained highly conserved across genotypes, as orthologous relationships were established using OrthoFinder (Emms & Kelly, 2019).

To verify PAVs and CNVs, genomic coordinates for each gene were extracted based on annotations (Table S1; Supporting Information S2) and were used for BLASTn/2.13.0 (where BLASTn is basic local alignment search tool, nucleotide) (Camacho et al., 2009) searches across all 76 genome assemblies (Jayakodi et al., 2024). The resulting genomic DNA sequences were retrieved using SAMtools/1.16.1 (H. Li et al., 2009) and aligned with annotation‐based coding sequences from all 76 genotypes. Sequence alignments were performed using MUSCLE (Edgar, 2004) in AliView (Larsson, 2014) and visualized with the R package ggmsa (Zhou et al., 2022). For newly identified sequences or copies, gene names and orthogroups were assigned based on genomic coordinates if annotations were available. Since *VRN2‐Hb* and *VRN2‐Hc* were absent from annotations in all 76 genotypes, all *VRN2*‐related targets were classified according to their reference sequences: AY485977.1 (*VRN2‐Ha*), AY485978.1 (*VRN2‐Hb*), and AY687931.1 (*VRN2‐Hc*) (Dubcovsky et al., 2005; Yan, Loukoianov, et al., 2004). The open reading frames of *VRN2‐Hb* and *VRN2‐Hc* were predicted using AUGUSTUS web interface (Stanke et al., 2006).

### 2.2 Analysis of transcript levels

We selected five representative genotypes (Akashinriki, Golden Promise, HOR 10350, Igri, and FT11) for functional analysis. Seeds were sown directly in soil and placed in a growth chamber under long‐day (LD) photoperiod conditions (16 h light/8 h dark) with day/night temperatures of 20°C/16°C. Three weeks after sowing, leaf samples were harvested at Zeitgeber time (ZT) 12 and ZT20, with three to four biological replicates per genotype per timepoint. Total RNA was extracted using the RNeasy Mini Kit (QIAGEN), and following a dsDNase treatment, cDNA was synthesized with the Maxima H Minus First Strand cDNA Synthesis Kit (Thermo Fisher Scientific). Quantitative real‐time‐polymerase chain reaction (qRT‐PCR) was performed on a QuantStudio 5 Real‐Time PCR System (Thermo Fisher Scientific) using PowerUp SYBR Green Master Mix (Thermo Fisher Scientific). Target gene expression was normalized to *ACTIN* (Campoli, Drosse, et al., 2012). Primer sequences were slightly adjusted to account for single nucleotide polymorphisms and/or 1 bp insertion/deletion (indel), ensuring gene‐ and genotype‐specific amplification (Table S2).

Barley pan‐transcriptome data, quantified as transcripts per million, for all five tissues were obtained from the barley genotype‐specific reference transcript datasets (RTDs; Guo et al., 2025). Due to inconsistencies between pan‐transcriptome gene IDs and pangenome gene (or orthogroup) IDs, gene IDs were linked based on genomic coordinates. Genes aligned with multiple truncated transcripts were excluded from the dataset. Transcripts of all CCT genes were extracted from 20 PBGv1 genotypes (Supporting Information S3), including *HvCO1* transcripts that aligned with two additional neighboring genes (Figure S1). Average transcript levels were calculated based on biological replicates (*n* = 2–3) and visualized using the ComplexHeatmap package (Gu et al., 2016). *VRN1* transcripts were extracted from shoot and inflorescence tissues and visualized using the R package ggplot2 (Wickham et al., 2017). Morex *VRN1* (*HORVU.MOREX.PROJ.5HG00452020*) transcripts were absent from the datasets.

To examine the transcriptome data used for *HvCO1* gene annotation, genotype‐specific RTDs, derived from RNA‐seq and Iso‐seq data, along with exon annotations, were extracted from Morex and Akashinriki (Guo et al., 2025). Additionally, Bowman transcriptome data from a diurnal cycle (Müller et al., 2020) were obtained to assess the circadian clock's influence on *HvCO1* expression and annotation. Raw reads from ZT04 (ERR3564268 and ERR3564269) and ZT16 (ERR3564258 and ERR3564259) were adapter‐trimmed using fastp/0.20 (Chen et al., 2018), aligned to the Akashinriki genome with STAR/2.7.9 (Dobin et al., 2013), and visualized using integrative genomics viewer (Robinson et al., 2011).

### 2.3 Phylogenetic analysis

While validating PAVs and CNVs, novel frameshift and/or premature stop variants were identified but excluded from phylogenetic analysis to prevent potential biases. However, newly identified copies of HvCMF4L and VRN2 were included. Based on full‐length protein sequences, sequence alignments were conducted using MAFFT/7.490 (Katoh & Standley, 2013), and maximum likelihood phylogenetic analysis was performed with IQ‐TREE/2.2.2.6 (Minh et al., 2020), employing 1000 bootstrap replications. The best‐fit amino acid substitution model was selected based on the Bayesian information criterion before constructing the phylogenetic tree. Phylogenetic trees were then visualized and annotated using iTOL (Letunic & Bork, 2007).

### 2.4 Principal component analysis (PCA)

To analyze sequence variations among genotypes, PCA was performed using *k*‐mer frequency‐based representations of amino acid sequences. The sequences were grouped by genotype to construct a *k*‐mer (*k* = 3) frequency matrix. Biostrings (Pagès et al., 2021) was used to read and process amino acid sequences, extracting 3‐mer subsequences for each genotype and counting their occurrences. The union of all *k*‐mers across genotypes was compiled to define the matrix columns. To account for partial deletions, PAVs, and CNVs, each row in the matrix, representing a genotype, was normalized by the total *k*‐mer count for that genotype. PCA was then performed using the FactoMineR package (Lê et al., 2008) with the normalized *k*‐mer frequency matrix as input. The PCA scores were extracted, and individual genotypes were visualized in two‐dimensional space, with points colored according to their cosine squared (cos^2^) values, indicating their contributions to the principal components. Visualization was conducted using the factoextra package (Kassambara & Mundt, 2020), applying a blue‐to‐red gradient to represent the degree of contribution. Labels were adjusted to minimize overlap, ensuring clear depiction of clustering patterns.

### 2.5 Analysis of *VRN2* regional synteny

In the selected genotypes Igri, Akashinriki, HOR 7552, HOR 6620, and 10TJ18, genomic regions containing all identified *VRN2* copies were extracted from their respective genome assemblies using SAMtools/1.16.1 (H. Li et al., 2009) based on the coordinates. A phylogenetic tree was first generated and constructed (as described above) to determine the order of pairwise sequence comparisons. Pairwise alignments were performed using minimap2/2.24 (H. Li, 2018) with the “‐ax sr” setting to align short genomic regions between genotypes. The alignments were processed with SyRI/1.6 (Goel et al., 2019) to retain alignment information, and structural variations were identified for each pairwise comparison. Plotsr/0.5.2 (Goel & Schneeberger, 2022) was used to visualize the synteny relationships, with a window size of 1000 bp, and a distance threshold of 1000 bp. *VRN2* gene positions were annotated according to their genomic coordinates.

## 3 RESULTS

### 3.1 Identification of CCT motif genes in 76 barley pangenome assemblies

**FIGURE 1:**
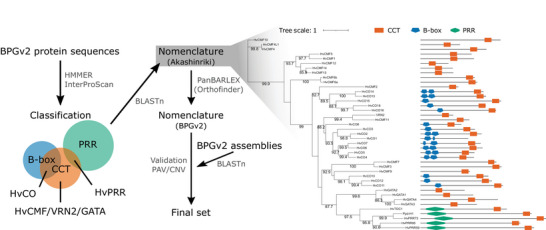
CONSTANS, CONSTANS‐LIKE, TIMING OF CAB EXPRESSION1 (CCT) motif proteins in barley and the identification pipeline. The identification process began with motif searches against the barley pangenome version 2 (BPGv2) protein sequences, followed by classification. Nomenclature was determined across all 76 genotypes based on orthologous relationships. Presence/absence variations (PAVs) and copy number variations (CNVs) were directly validated from the BPGv2 assemblies. The phylogenetic tree represents the identified candidates (prior to validation) in Akashinriki, including their expected domains, and was constructed using the maximum likelihood IQ‐TREE JTT+F+I+G4 model with 1000 bootstrap replications (values ≥ 80 are displayed). PRR, pseudo‐response regulator.

To systematically identify all CCT motif genes, we employed a conventional motif search approach using the BPGv2 protein sequence database (panbarlex.ipk‐gatersleben.de; Jayakodi et al., 2024). HvCO and HvPRR candidates were classified based on the overlap between identified CCT motif hits and corresponding B‐box or PRR motif hits, respectively (Figure 1). In addition to the expected HvCMF and VRN2, the identified CCT motif‐only candidates also include members of the GATA family of transcription factors, which are conserved in Arabidopsis and rice (Reyes et al., 2004). For nomenclature and validation of the motif‐classified sets, we compared the identified sequences with previously published data (Campoli, Drosse, et al., 2012; Cockram et al., 2012; Griffiths et al., 2003). We selected the winter barley cultivar Akashinriki as the reference for nomenclature, as some candidates, including VRN2 and HvPRR59, were already undetected in Morex at this stage (Supporting Information S1). Each candidate in Akashinriki was assigned a name according to its corresponding reference. Interestingly, contrary to the expectation of multiple copies (Dubcovsky et al., 2005), only a single copy of VRN2 was detected. Additionally, Akashinriki HvCO1 was recovered from a B‐box motif‐only candidate, suggesting a possible CCT motif deletion (Figure 1). Nevertheless, the phylogenetic relationships largely aligned with the findings in Morex (Huang et al., 2023), with the addition of the nomenclature for HvCMF2, HvCMF9, HvCMF12, and HvCMF14. The assigned names were then propagated across all 76 genotypes based on established orthologous relationships (i.e., orthogroup ID, panbarlex.ipk‐gatersleben.de; Jayakodi et al., 2024).

**FIGURE 2:**
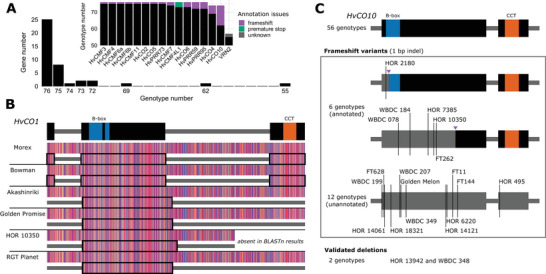
Validation of presence/absence variations (PAVs) in barley CONSTANS, CONSTANS‐LIKE, TIMING OF CAB EXPRESSION1 (CCT) motif genes. (A) PAVs of CCT motif genes across 76 barley genotypes. Twenty‐five genes were present in all 76 genotypes, while the PAVs of the remaining genes were validated and characterized, with potential annotation issues highlighted (inner plot). (B) Reading frame variations of annotated *HvCO1* across selected genotypes, leading to potential deletions in the CCT motif. Nucleotides are color‐coded according to the "Zappo" scheme in the sequence alignment. For each genotype, annotated coding regions are shown as black boxes beneath the corresponding genomic region. (C) Frameshift variants and deletions identified in *HvCO10*. Purple arrows indicate alternative translation start sites. Morex was used as a reference for the gene models in (B) and (C).

The conservation of each candidate across the 76 genotypes allowed the classification of genes as core or dispensable within the pangenome context (Marroni et al., 2014). Of the 41 genes, 25 were present in all 76 genotypes and were considered as core genes, likely responsible for the major phenotypic traits (Figure 2A). The remaining genes were categorized as dispensable exhibiting greater diversity and potentially contributing to phenotypic plasticity. Thirteen of these dispensable genes were present in at least 72 genotypes, while *HvCO4*, *HvCO10*, and *VRN2* were found in only 69, 62, and 55 genotypes, respectively.

### 3.2 Validation of PAVs and CNVs in barley CCT motif genes

Although protein databases and gene annotation‐based methods are widely used for gene family identification, even in the pangenome era (Tong et al., 2025), careful validation of PAVs and CNVs remains crucial to prevent annotation errors, particularly when annotations rely on projections (Jayakodi et al., 2024). To verify the variations in the candidates identified so far, such as deletion of the CCT domain in Akashinriki HvCO1 and the absence of the expected VRN2 copies, we conducted a systematic search of all candidates using their genomic DNA sequences directly against the genome assemblies (Figure 1; Supporting Information S2).

While identified in all 76 genotypes, many core genes, including *HvCO1*, exhibited potential coding frame variations. For example, genotypes such as Golden Promise, HOR 10350, and RGT Planet, showed CCT domain deletions in HvCO1 at the protein level, similar to Akashinriki (Figure 2B). In these genotypes, the first and last exons of *HvCO1* were not transcribed and/or annotated compared to Morex and Bowman, leading to the loss of the CCT domain while retaining intact B‐box domains. However, this may be due to annotation mistakes, as the genomic DNA sequences of *HvCO1* remain intact and highly conserved across these genotypes. The genotypes presented as examples here were all part of the first version of the barley pangenome (BPGv1), where gene annotations were primarily based on transcript evidence (Guo et al., 2025; Jayakodi et al., 2024). However, the annotated exons in both Morex and Akashinriki did not align with exons supported by RNA‐seq and Iso‐seq data (Figure S1A), suggesting that the annotated coding regions may have been derived mainly from projections, potentially leading to inaccuracies.

The absence of *HvCO1* in the pan‐transcriptome RNA‐seq and Iso‐seq data may be attributed to reliance on a single harvesting timepoint that missed its expression window. Analysis of Bowman's diurnal transcriptome (Müller et al., 2020) revealed that *HvCO1* exhibited diurnal expression patterns, peaking in the late day and evening (Figure S1B). We selected ZT04 (low expression) and ZT16 (high expression) and mapped RNA‐seq reads to the Akashinriki genome. Notably, the unannotated CCT coding exon 3 in Akashinriki (identical to Morex) showed coverage enrichment exclusively at ZT16, supporting its misannotation due to insufficient sampling timepoint failing to capture its active expression phase. To assess the functionality of these unannotated *HvCO1* regions, we performed qRT‐PCR on Akashinriki and Golden Promise leaf tissues at ZT12 and ZT20. *HvCO1* expression was detectable at both timepoints (Figure S2A), consistent with prior findings (Zahn et al., 2023), suggesting the CCT coding regions may remain functional.

Similar coding frame variations were observed in other CCT motif genes, including *HvCMF1*, *HvCMF3*, *HvCMF4L1*, *HvCMF10*, *HvCO2*, *HvCO18*, *HvGATA4*, and *HvPRR59* (Table S1). Moreover, the extended open reading frame observed in HOR 10350 may result from frameshift insertions, whereas the deletion of the CCT domain was validated by the absence of the corresponding coding region, suggesting a true loss of this domain.

In addition to validating the identified candidates, our approach uncovered novel candidates that were undetectable at the protein level (Figure 2A, inner plot; Table S1). Many of these were absent due to frameshift mutations and/or premature stop codons, particularly in the case of *HvCO10*, where 12 genotypes lacked annotations and two exhibited true genomic deletions (Figure 2C). Furthermore, six genotypes with annotated *HvCO10* were also affected by frameshift mutations (1 bp indel). However, alternative translation start sites allowed them to remain in frame: In HOR 2180, this resulted in a partial deletion of the B‐box domain, while in the other five genotypes, approximately half of the reading frame, including the CCT domain, was retained. Expression analysis of *HvCO10* in selected genotypes (full‐length, truncated, and unannotated variants) revealed that CCT domain‐containing transcripts remained detectable in frameshift variants (Figure S2B), suggesting potential functionality of truncated proteins.

Meanwhile, among the unannotated genotypes, HOR 14121, HOR 6220, FT11, FT144, and HOR 495 were predicted to contain only the B‐box domain, highlighting *HvCO10* as one of the most diverse CCT genes with extensive natural mutations. In contrast, similar natural variants were identified in only a few genotypes of other CCT motif genes (Figure 2A; Table S1).

**FIGURE 3:**
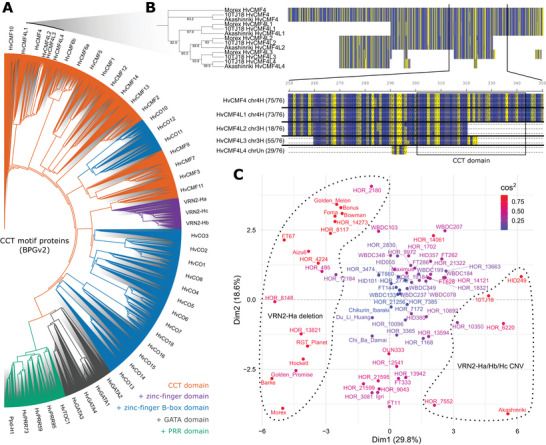
Diversity of CONSTANS, CONSTANS‐LIKE, TIMING OF CAB EXPRESSION1 (CCT) motif proteins in the barley pangenome. (A) Phylogeny of CCT motif proteins across 76 genotypes. The phylogenetic tree was constructed using the maximum likelihood IQ‐TREE JTT+F+I+G4 model. Branches are colored based on the presence of CCT domain alone (except for HvCMFL4) or the addition of other domains. (B) Phylogeny of HvCMF4 and HvCMF4L in selected genotypes. The phylogenetic tree was constructed using IQ‐TREE JTT+I model with 1000 bootstrap replications (values ≥ 80 are displayed). Amino acids are color‐coded according to the "Strand" scheme in the sequence alignment. The CCT domain is based on Morex HvCMF4. The number in brackets next to the sequence alignment indicates the number of genotypes containing the corresponding annotated copy. (C) Principal component analysis (PCA) of CCT motif protein variation across the barley pangenome. PCA was based on all CCT motif proteins as shown in (A), grouped by genotype. Cosine squared (cos^2^) values reflect the contribution of each genotype to the principal components. The genotype clusters, marked by dashed lines, are based VRN2‐Ha deletion and VRN2‐Ha/Hb/Hc copy number variations, manually assigned according to Table S3. PRR, pseudo‐response regulator.

We then investigated genes expected to have additional paralogs. Previous phylogenetic analysis indicated that barley (Morex) contained *HvCMF4* paralogs, *HvCMF4L1* (also known as *HvCMF8*) and *HvCMF4L2*, which were reported to have partially redundant roles in spikelet development regulation (Huang et al., 2023). While only HvCMF4 and HvCMF4L1 were detected at the protein level using motif searches (Figures 1 and 2A), we successfully detected *HvCMF4L2* and two additional copies by searching with *HvCMF4L1* genomic DNA sequences. To validate the phylogenetic relationships of these newly identified copies, we constructed a phylogenetic tree including all CCT motif proteins, excluding frameshift variants (Figure 3A). As expected, the new copies clustered together with HvCMF4 in the same clade and were designated as HvCMF4L3 and HvCMF4L4. Sequence analysis revealed that HvCMF4L2 and HvCMF4L3 exhibited partial deletions of the CCT domain (Figure 3B), which explained why they were not initially detected through motif searches. In contrast, HvCMF4L4 lacked the entire CCT domain and shared only partial sequence conservation with HvCMF4, HvCMF4L2, and HvCMF4L3, excluding it from being classified as a CCT motif protein. HvCMF4L2 was identified in only 18 genotypes, while the newly discovered HvCMF4L3 was more abundant, present in 54 genotypes. Both genes were dispensable and located on chromosome 3H at different positions, suggesting that their presence was not due to intrachromosomal translocation, as supported by limited sequence conservation and the co‐occurrence of both paralogs in certain genotypes, such as 10TJ18. Consistent with previous findings (Huang et al., 2023), these results indicated that HvCMF4L1, HvCMF4L2, and HvCMF4L3 likely arose from independent duplication events of HvCMF4.

As the most “dispensable” CCT motif gene present in the “shell pangenome” of barley, *VRN2* is expected to exhibit multiple copies, referred to as *VRN2* (or *ZCCT*)*‐Ha*/*Hb*/*Hc* (Dubcovsky et al., 2005; von Zitzewitz et al., 2005). These copies encode proteins containing a CCT domain, with VRN2‐Ha and VRN2‐Hb also possessing an additional zinc‐finger domain. While only a single copy of VRN2 was annotated in the barley pangenome (Figure 1), additional copies were identified by searching genome assemblies using published reference sequences (Yan, Loukoianov, et al., 2004). In total, 57 genotypes were found to contain at least one *VRN2* copy (Figure 2A; Figure S3A; Table S3). However, as the annotation of these copies was not supported by pan‐transcriptome data (Figure S4), their reading frames were predicted and subsequently classified and validated through phylogenetic analysis (Figure 3A; Figure S3A).

### 3.3 Refined phylogeny of CCT motif proteins in barley

With the inclusion of the missing copies, we established a comprehensive phylogenetic overview of CCT motif proteins at the barley pangenome level (Figure 3A). Despite observed genotypic variations (e.g., partial deletions; Figure 2B,C), each CCT member distinctly grouped into separate clades, reinforcing their correct nomenclature based on orthologous relationships.

The pangenome phylogeny identified HvCMF10, HvCMF4, and HvCMF4L as the most ancestral CCT family members, consistent with single‐genotype analyses of MorexV2 (Huang et al., 2023) and Akashinriki (Figure 1). In the pangenome tree, HvCMF6a and HvCMF6b emerged as the next diverged paralogs (Figure 3A). However, Akashinriki‐specific phylogeny revealed them diversified subsequently, after HvCMF5, HvCMF1, HvCMF12, HvCMF14, and HvCMF13 (Figure 1), highlighting how pangenome analysis reduces biases from genotype‐specific variations. The BPGv1 pan‐transcriptome data (Guo et al., 2025) revealed strong *HvCMF6a* and *HvCMF6b* expression in caryopsis, with *HvCMF6a* also expressed in inflorescence (Figure S4; Supporting Information S3). While *HvCMF4* transcripts were generally low or undetectable, *HvCMF4L2* showed strong inflorescence‐specific expression in genotypes such as HOR 10350, HOR 9043, and Morex. The other *HvCMF4L* genes remained below the detection threshold across all samples.

The first B‐box domain addition within the CCT family coincided with a major divergence into two clades: one consisting of HvCO12, HvCO11, and HvCO10 (single B‐box), and the other containing HvCMF9, HvCMF3, and HvCMF7 (no B‐box; Figure 3A). Notably, the B‐box‐containing clade was phylogenetically distant from other HvCOs, some of which possessed two B‐box domains. HvCMF3 and HvCMF7 clustered within the second clade, with *HvCMF3* showing high expression in shoots, while *HvCMF7* was broadly expressed across all five tissues (Figure S4; Supporting Information S3).

Similarly, the addition of the zinc‐finger domain in VRN2‐Ha was an independent evolutionary event that occurred during its diversification from HvCMF11 (Figure 3A). However, VRN2‐Hb and VRN2‐Hc appeared to have arisen through duplications of VRN2‐Ha, with VRN2‐Hc subsequently losing the zinc‐finger domain. This was supported by the observations that all genotypes carrying VRN2‐Ha also contained VRN2‐Hb and VRN2‐Hc, except for the wild barley (*Hordeum spontaneum* (K. Koch) Thell.) genotype FT67, which only possessed VRN2‐Hb and VRN2‐Hc (Table S3).

Most of the remaining HvCOs have unknown functions, except for HvCO1 and HvCO2, which are conserved across species for their role in regulating flowering time (Campoli, Drosse, et al., 2012). They each contained an additional B‐box domain compared to their putative ancestor HvCO3 (Figures 1 and 3A), suggesting domain duplication contributed to functional specialization.

The most recently evolved CCT members acquired zinc‐finger GATA or PRR domains. Four barley GATA factors showed distinct expression patterns: *HvGATA2* was predominantly expressed in roots, *HvGATA1* was specific to inflorescences, while *HvGATA3* and *HvGATA4* exhibited overall higher expression levels particularly in inflorescences (Figure S4; Supporting Information S3). Among PRR family members (HvTOC1, HvPRR95, HvPRR59, HvPRR73, and Ppd‐H1), we observed high *Ppd‐H1* expression in shoots and inflorescences and *HvTOC1* predominance in roots (Figure S4; Supporting Information S3).

The refined phylogenetic analysis of CCT motif proteins in the barley pangenome revealed distinct clades, domain expansions, and potential functional divergences. However, the impact of genotypic variation remained unclear, as all genotypes clustered within the same clade for each CCT motif protein, despite the variations described above. To address this, we performed PCA on the same set of sequences (all CCT proteins) separated by genotypes. The PCA plot revealed that most genotypes, regardless of row‐type or geographic origin, clustered together, indicating minimal contribution to variation (Figure 3C). In contrast, genotypes with stronger contributions were primarily separated by Dim1 (29.8%), driven largely by PAVs and CNVs of *VRN2* (Table S3), consistent with *VRN2* being identified as the most dispensable CCT motif gene in barley (Figure 2A).

To determine whether this PCA‐based approach could reliably identify the most variant genes and to examine the role of Dim2 (18.6%) in separating the VRN2‐Ha deletion cluster, we repeated the analysis excluding all VRN2‐related sequences. Notably, genotypes previously separated by Dim2 were now separated along Dim1 (29.2%) in the new PCA plot (Figure S5A). To further explore this separation, we selected two sets of highly contrasting genotypes—Morex and Barke versus HOR 2180 and Bowman—and constructed a phylogenetic tree (Figure S5B). This analysis pinpointed HvCMF1 as the driver of the observed separation, which aligned with the presence of two major reading frame variants in HvCMF1 based on annotation (Figure S5C; Table S1). However, it is important to note that this could also be influenced by potential annotation errors, as previously observed in HvCO1 (Figure S1), necessitating further validation.

### 3.4 Barley pangenome insights into *VRN2* and growth habit variation

The 76 genotypes in BPGv2 represented diverse growth habit, including 38 spring barleys, 16 winter barleys, 10 facultative types (which display spring‐like growth without vernalization requirements but retain strong low‐temperature tolerance; von Zitzewitz et al., 2011), and 12 uncharacterized genotypes (Table S3). While most genotypes with VRN2 deletions were well‐characterized spring barley cultivars (e.g., Morex, Barke, and Golden Promise), winter barley genotypes such as Aizu6, HOR 4224, and HOR 12184 also exhibited complete VRN2 deletions. Surprisingly, several spring and facultative barley genotypes retained intact VRN2‐Ha/Hb/Hc copies, with HOR 6220 and HOR 7552 even carrying additional copies.

**FIGURE 4:**
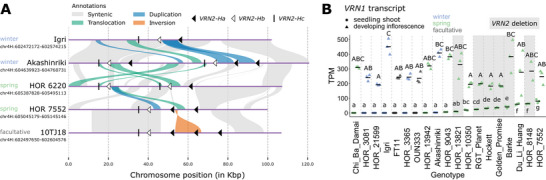
The presence of *VRN2* is not associated with winter growth habit in barley. (A) Synteny of genomic regions containing *VRN2* copy number variations in selected genotypes, including winter barleys Akashinriki and Igri, spring barleys HOR 6220 and HOR 7552, and the facultative barley genotype 10TJ18. *VRN2* copies are indicated based on their coordinates, located near regions with complex duplications, translocations, and inversion. (B) *VRN1* transcript levels in seedling shoots and developing inflorescences across 19 barley genotypes. Horizontal bars represent mean values from biological replicates (*n* = 2–3). Different letters indicate significant differences (lowercase and uppercase letters for seedling shoots and developing inflorescences, respectively, one‐way analysis of variance (ANOVA) and Tukey's honestly significant difference (HSD) test, *p* < 0.05). Genotypes shaded in gray have deletions of all *VRN2* copies. Growth habit information in (A) and (B) is based on Jayakodi et al. (2024). TPM, transcripts per million.

Since all identified *VRN2* copies were located in close proximity on chromosome 4H, we selected representative genotypes to validate CNVs and local chromosomal structures. Although both Igri and Akashinriki are winter barley cultivars, Akashinriki carried two additional *VRN2‐Ha* copies, and an extra copy of *VRN2‐Hb* and *VRN2‐Hc* (Figure 3C; Table S3), likely resulting from regional duplication and/or translocation (Figure 4A). Compared to Akashinriki, the spring barley landrace HOR 6220 contained only one *VRN2‐Ha* copy, similar to Akashinriki's second copy, while *VRN‐Hb* and *VRN2‐Hc* regions underwent complex translocations and duplications. Even between spring barley genotypes, *VRN2* CNVs were not entirely syntenic between HOR 6220 and HOR 7552. Similar to spring and winter barleys, facultative barleys in the pangenome displayed *VRN2* PAVs and CNVs (Figure 3C; Table S3). For instance, the additional *VRN2‐Ha* copy in 10TJ18, compared to HOR 7552, likely resulted from local inversion and insertion (Figure 4A).

Expression analysis of *VRN2* homologs revealed that *VRN2‐Ha* and *VRN2‐Hb* showed high expression levels in winter barleys (Akashinriki and Igri), detectable but reduced in facultative and spring barleys (FT11 and HOR 10350), and absent in *VRN2*‐deleted genotypes like Golden Promise (Figure S3B). However, no *VRN2‐Hc* transcripts were detected in any genotypes at either timepoints.

To investigate the regulatory roles of *VRN2* PAVs and CNVs in growth habit determination, we analyzed *VRN1* expression patterns using pan‐transcriptome data (Guo et al., 2025). Comparing *VRN1* transcript levels in seedling shoots (pre‐vernalization) and developing inflorescences (post‐vernalization) revealed genotype‐specific vernalization responses: Winter barleys showed minimal *VRN1* pre‐vernalization but strong induction post‐vernalization, confirming their vernalization sensitivity (Figure 4B). In contrast, spring barleys typically exhibited higher *VRN1* transcript levels before vernalization, consistent with vernalization independence. However, exceptions were observed in some spring or facultative genotypes, such as Chi Ba Damai, FT11, OUN333, HOR 13942, and HOR 9043, which displayed winter‐like *VRN1* repression, potentially linked to functional *VRN2* copies (Figure 3C; Table S3). This aligned with higher *VRN2* expression in FT11 compared to HOR 10350, while the latter exhibited significantly higher *VRN1* levels (Figure S3B). Paradoxically, other *VRN2*‐containing spring genotypes, such as Du Li Huang and HOR 7552, maintained high *VRN1* expression, raising questions about the exact repression role of *VRN2* in barley flowering regulation. Collectively, our pangenome analysis revealed that *VRN2* structural variation did not strictly correlate with growth habit in barley, suggesting the need for pangenome‐assisted studies to dissect the interplay between *VRN2* diversity, genomic context, and vernalization responses.

## 4 DISCUSSION

Advancements in plant pangenomics have revolutionized the study of gene families, offering unprecedented opportunities to explore genetic diversity, evolutionary dynamics, and functional variation within and across species (Jayakodi et al., 2025; Tao et al., 2019). However, transitioning from single‐reference genome analyses to pangenome‐based frameworks necessitates careful methodological considerations to ensure accurate and comprehensive gene family characterization. Our study highlights the importance of integrating multiple data sources and validation strategies to overcome the limitations of traditional approaches, particularly in structurally complex regions and highly variable gene families such as barley CCT motif genes.

Accurate detection of PAVs and CNVs is pivotal in pangenome‐based gene family analysis. The examination of CCT motif proteins within the barley pangenome has revealed notable gene annotation discrepancies, especially concerning core genes like *HvCO1*. These discrepancies suggest that the observed variations may result from annotation inaccuracies rather than genuine genetic differences. Current gene annotation strategies, including those employed in the barley pangenome (Jayakodi et al., 2024), rely on both transcript evidence and projection methods. While pan‐transcriptome data offer genotype‐specific evidence across five tissues (Guo et al., 2025), they are limited by factors such as sampling at a single time point, which may lead to missing information in the transcriptome data of CCT genes, thereby shifting annotations toward projections. A significant portion of the barley transcriptome (∼84%) is considered rhythmic and is regulated by the circadian clock under LD conditions (Müller et al., 2020). Although the diurnal expression patterns of the major flowering regulator *HvCO1* are well‐documented (Campoli, Drosse, et al., 2012; Zahn et al., 2023) and account for its misannotation in the barley pangenome, some tissue‐ and/or stress‐specific genes may still be incorrectly annotated due to insufficient transcript data. These findings highlight the need for careful validation of gene annotations, integrating comprehensive transcriptomic and genomic data to accurately define core genes and their functional domains.

While motif‐based searches can identify core CCT motif genes, direct examination of genome assemblies has uncovered potentially functionally disruptive frameshift mutations and domain deletions, such as those in *HvCO10*. Notably, despite these structural changes, transcripts containing the CCT domain remained detectable, suggesting potential functional implications. These findings demonstrate that relying solely on protein‐level annotations can overlook structural variants, particularly in genes under positive selection pressures that exhibit high intraspecies diversity. Such novel natural variants, however, represent valuable resources for functional studies. For instance, the diverse functions of the barley flowering time regulator *EARLY FLOWERING 3* have been elucidated through natural allelic variation (Huang et al., 2024; Zahn et al., 2023; Zhu et al., 2023). To determine whether these HvCO10 variants contribute to proteome complexity—particularly given the persistence of CCT domain transcription—large‐scale mass spectrometry–based proteomic analyses would be essential (Tress et al., 2017).

The pangenome approach proved particularly valuable for phylogenetic analysis of CCT proteins, as it minimized biases from genotypic variation while revealing deep evolutionary patterns. Notably, the divergence of HvCMF6a/b from HvCMF4/HvCMF4L was detectable only in the pangenome phylogeny. Their high expression levels in caryopses and inflorescences suggest roles in inflorescence development and grain filling, potentially analogous to *HvCMF4*/*HvCMF4L* functions (Huang et al., 2023). Domain architecture analysis indicated functional divergence, particularly in clades that acquired additional B‐box domains, likely through independent evolutionary events. While the CCT domain facilitates DNA binding and B‐box domains mediate protein–protein interactions (Ben‐Naim et al., 2006), the consequences of these domain combinations remain unclear. The clustering of HvCMF3 and HvCMF7 aligns with their conserved roles in chloroplast development (M. Li et al., 2019, 2021), though whether this function predates their divergence from HvCMF9 is unknown. If ancestral, subsequent B‐box domain additions in HvCO10/11/12 may have modified this functionality. Future cross‐species pangenome comparisons could help determine whether these B‐box domain acquisitions represent conserved patterns across Poaceae, and whether they correlate with specific functional adaptations in different lineages.

Among GATA transcription factors (widespread eukaryotic DNA‐binding proteins; Lowry & Atchley, 2000), we identified four members in barley, homologous to Arabidopsis class C GATA factors, characterized by TIFY and CCT motifs but with unclear functions (reviewed in Schwechheimer et al., 2022). These likely represent secondary CCT family additions, lacking HvCMF homology while possibly retaining ancestral GATA‐related roles supplemented by CCT domains. A parallel pattern may occur in PRR family members: Although most of them have conserved circadian functions (Müller et al., 2020), Ppd‐H1 appears to acquire its circadian role in barley after diverging into AtPRR3/7 in Arabidopsis (Turner et al., 2005). Consistent with its photoperiod response function (Campoli, Shtaya, et al., 2012), *Ppd‐H1* showed high expression in shoots and inflorescences, suggesting its primary photoperiod role was later co‐opted into the circadian network. Conversely, root‐predominant *HvTOC1* expression implies its role in specialized circadian regulation in root tissues.

The identification of intact *VRN2* copies in spring and facultative barley genotypes challenges the canonical model wherein vernalization requirements are governed exclusively by *VRN2* deletions (Landis et al., 2024; Yan, Loukoianov, et al., 2004). This unexpected observation prompts two key questions: whether *VRN2* fulfills alternative biological roles beyond vernalization repression, and whether compensatory mechanisms involving other repressors, such as the wheat (*Triticum aestivum* L.) MADS‐box transcription factor gene *ODDSOC2* (Dixon et al., 2019; Greenup et al., 2010; Jacott & Boden, 2020), contribute to barley's vernalization response. While temperate cereals like wheat and barley employ a *VRN1*‐centric pathway distinct from Arabidopsis’ *FLC*‐dependent mechanism (reviewed in Xu & Chong, 2018), our transcript analyses revealed no clear correlation between *VRN1* expression and growth habit or *VRN2* deletions. This inconsistency suggests more complex regulation, potentially mediated by *VRN1* allelic variation, supported by the identification of its intronic and/or promoter indels possibly modulating its pre‐vernalization expression (Hemming et al., 2009; von Zitzewitz et al., 2005; Yan, Helguera, et al., 2004). Notably, barley's diploid genome may render its flowering network more sensitive to genetic variation compared to polyploid wheat, where *VRN1* haplotypes in subgenome A alone determine growth habit (Jiao et al., 2024). These observations from the recent wheat pangenome study underscore the need for similar investigations in the barley pangenome to unravel species‐specific regulatory mechanisms.

The inconsistency between *VRN1* transcript levels and growth habit may also reflect limitations in current growth habit classification systems. Growth habit is typically assessed through vernalization requirements and/or sensitivity, which are quantitative traits approximated by measuring the days to flowering in nonvernalized plants. This approach may not align strictly with binary winter or spring classifications. For instance, genotypes with complete or partial *VRN2* deletions can still exhibit varying degrees of vernalization sensitivity, intermixed with genotypes containing intact *VRN2* copies (Muñoz‐Amatriaín et al., 2020). The most dramatic example is the wild barley FT11 in this study, which was classified as both spring (Jayakodi et al., 2020) and facultative (Jayakodi et al., 2024), despite possessing functional *VRN2* copies and *VRN1* transcript profiles similar to winter barleys. These inconsistencies highlight the need for more rigorous, experimentally derived phenotyping criteria to properly distinguish between growth habit categories.

In addition to genetic factors, the “winter memory” mechanism underlying vernalization is epigenetically regulated. In wheat and barley, the upregulation of *VRN1* during vernalization is associated with a shift in histone modification marks, transitioning from the repressive H3K27me3 to the activating H3K4me3 (Diallo et al., 2012; Oliver et al., 2009). Advanced epigenomic profiling techniques have mapped chromatin states of key vernalization genes both before and after vernalization, uncovering vernalization‐related regulatory elements in wheat, including distal accessible chromatin regions upstream of *FT* (Y. Liu et al., 2024). The identification of these regulatory elements, which requires functional genomic annotations (i.e., by epigenomic profiling), were not covered in the current analysis of CCT genes.

The current set of barley pangenome genotypes, featuring high‐quality assemblies and verified *VRN2* variations, offers an excellent starting point to revisit and refine the definitions of growth habit and vernalization mechanisms in barley.

## AUTHOR CONTRIBUTIONS

**Zihao Zhu**: Conceptualization; data curation; formal analysis; investigation; methodology; visualization; writing—original draft. **Nils Stein**: Conceptualization; funding acquisition; writing—review and editing.

## CONFLICT OF INTEREST STATEMENT

The authors declare no conflicts of interest.

## Supporting information




**Figure S1**. Absence of transcriptome support for *HvCO1* annotation. (A) Genomic regions containing *HvCO1* (grey shaded area) in Morex and Akashinriki, showing gene annotations (Jayakodi et al., 2024), genotype‐specific reference transcript datasets (RTDs) from RNA‐seq and Iso‐seq, and exon annotations (Guo et al., 2025). For Akashinriki, additional RNA‐seq coverage and junction tracks were mapped using Bowman data (Bow2Aka, ZT04/ZT16). The unannotated exon 3 (identical to Morex) is marked by a red horizontal bar. (B) Diurnal expression patterns (CPM, counts per million) of *HvCO1* in Bowman, with arrows indicating the timepoints used for Akashinriki mapping in (A). Data were obtained from Müller et al. (2020).
**Figure S2**. Expression of *HvCO1* and *HvCO10* across genotypic variants. Transcript levels of *HvCO1* (A) and *HvCO10* (B) in selected genotypes. Leaf samples were harvested at ZT12 and ZT20. Expression levels were normalized to *ACTIN*. Horizontal bars represent mean values from biological replicates (n = 3–4).
**Figure S3**. Classification and expression of *VRN2*. (A) Phylogeny of *VRN2* across barley pangenome genotypes. The phylogenetic tree was constructed based on coding sequences using the maximum likelihood IQ‐TREE HKY+F+G4 model. *VRN2* copies are classified based on reference sequences (Yan et al., 2004b; Dubcovsky et al., 2005), which are highlighted with neon green branches. (B) Transcript levels of *VRN2* copies in selected genotypes. Leaf samples were harvested at ZT12 and ZT20. Expression levels were normalized to *ACTIN*. Horizontal bars represent mean values from biological replicates (n = 3–4). Golden Promise, shaded in grey, has deletions of all *VRN2* copies.
**Figure S4**. Expression variability of CCT genes across 20 barley genotypes. The heatmap illustrates the average transcript levels (n = 2–3), quantified in transcripts per million (TPM, Guo et al., 2025) for all CCT genes across five tissues. For each tissue, data from 20 genotypes are displayed in the following order (left to right): Akashinriki, Barke, Chi Ba Damai, Du Li Huang, FT11, Golden Promise, Hockett, HOR 10350, HOR 13821, HOR 13942, HOR 21599, HOR 3081, HOR 3365, HOR 7552, HOR 8148, HOR 9043, Igri, Morex, OUN333, and RGT Planet. The gene order is determined by the phylogenetic tree (Figure 3A). *HvCO1* transcripts, indicated by an asterisk, were aligned with two additional neighboring genes (Figure S1A).
**Figure S5**. HvCMF1 variation in the barley pangenome. (A) Principal component analysis (PCA) of CCT motif protein variation across the barley pangenome, excluding VRN2. Cosine squared (cos^2^) values reflect the contribution of each genotype to the principal components. Arrows highlight selected contrasting genotypes: Morex and Barke versus HOR 2180 and Bowman. (B) Phylogeny of CCT motif proteins, excluding VRN2, in selected genotypes. The phylogenetic tree was constructed using the maximum likelihood IQ‐TREE JTT+F+I+G4 model, with branches colored according to genotype groups, emphasizing HvCMF1 as the most divergent protein between groups. (C) Two major HvCMF1 variants identified in the barley pangenome, potentially explaining the genotypic separation observed in (A). Amino acids are color‐coded according to the ‘Strand’ scheme in the sequence alignment.

**Table S1**. Genomic validation of CCT genes.

**Table S2**. Oligonucleotide primers used in gene expression analysis.

**Table S3**. PAVs and CNVs of *VRN2* in the barley pangenome.

**Dataset S1**. Barley CCT motif proteins identified through HMMER search.

**Dataset S2**. Barley CCT genes identified by BLASTn.

**Dataset S3**. Transcriptome data of CCT genes across 20 barley genotypes.

## Data Availability

The data supporting the findings of this study are available within the article and its supporting information files. Additional datasets can be accessed at https://github.com/zihaozhu92/BPGv2‐CCT. This repository includes multiple sequence alignment files for phylogenetic analysis and visualization, raw phylogenetic tree files with bootstrap values, input sequences for synteny analysis, and scripts for principal component analysis and data visualization. Any further inquiries or requests can be directed to the corresponding author.
