# Supplementary material for: Pangenome insights into structural variation and functional diversification of barley CCT motif genes
Source: Plant Genome. 2025 Aug 26;18(3):e70098. doi: 10.1002/tpg2.70098 (PMC12381546; doi:10.1002/tpg2.70098)
Supplement: Supplementary file 1 [file TPG2-18-e70098-s003.pdf]

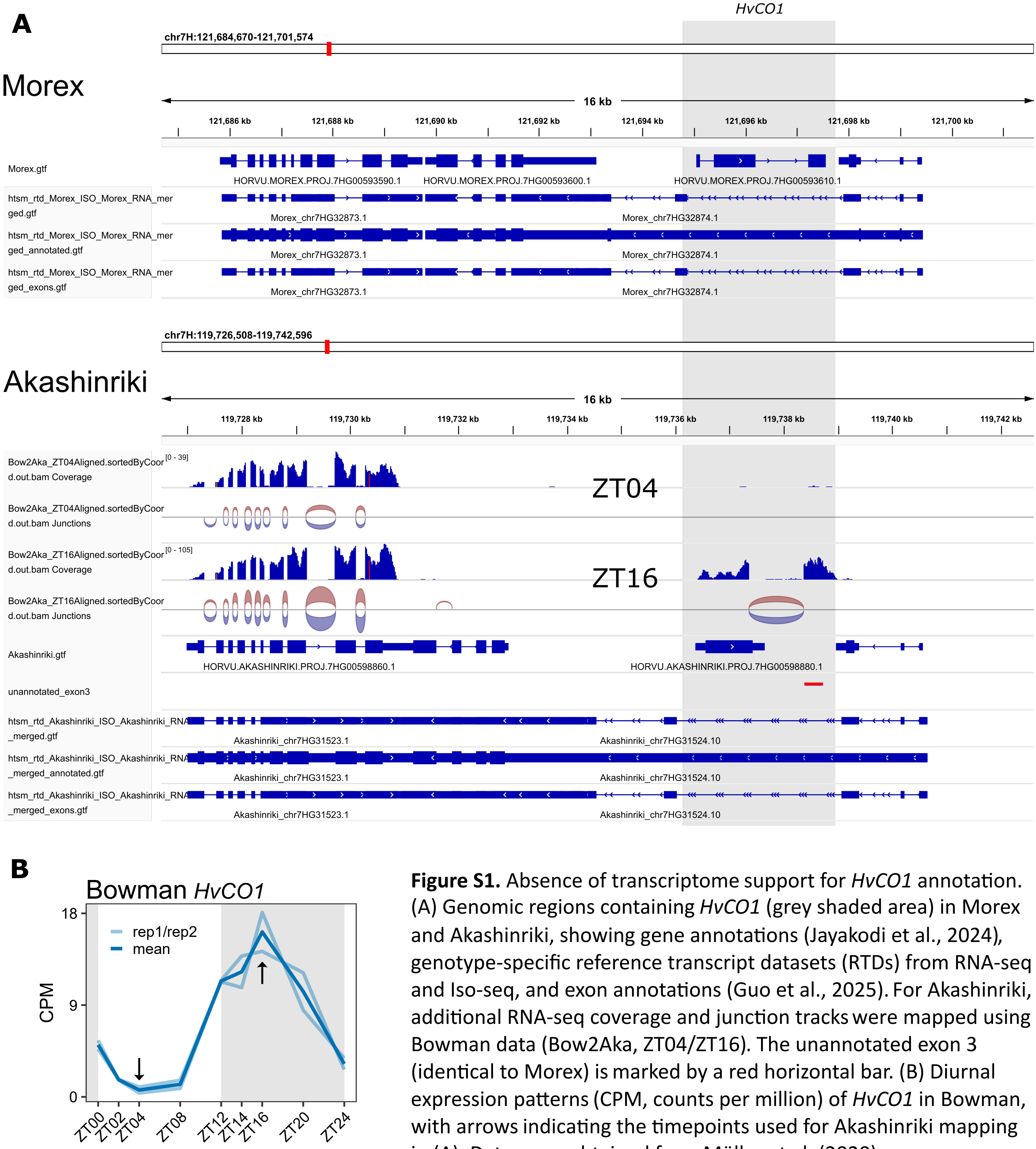

Figure S1. Absence of transcriptome support for *HvCO1* annotation. (A) Genomic regions containing *HvCO1* (grey shaded area) in Morex and Akashinriki, showing gene annotations (Jayakodi et al., 2024), genotype-specific reference transcript datasets (RTDs) from RNA-seq and Iso-seq, and exon annotations (Guo et al., 2025). For Akashinriki, additional RNA-seq coverage and junction tracks were mapped using Bowman data (Bow2Aka, ZT04/ZT16). The unannotated exon 3 (identical to Morex) is marked by a red horizontal bar. (B) Diurnal expression patterns (CPM, counts per million) of *HvCO1* in Bowman, with arrows indicating the timepoints used for Akashinriki mapping in (A). Data were obtained from Müller et al. (2020).

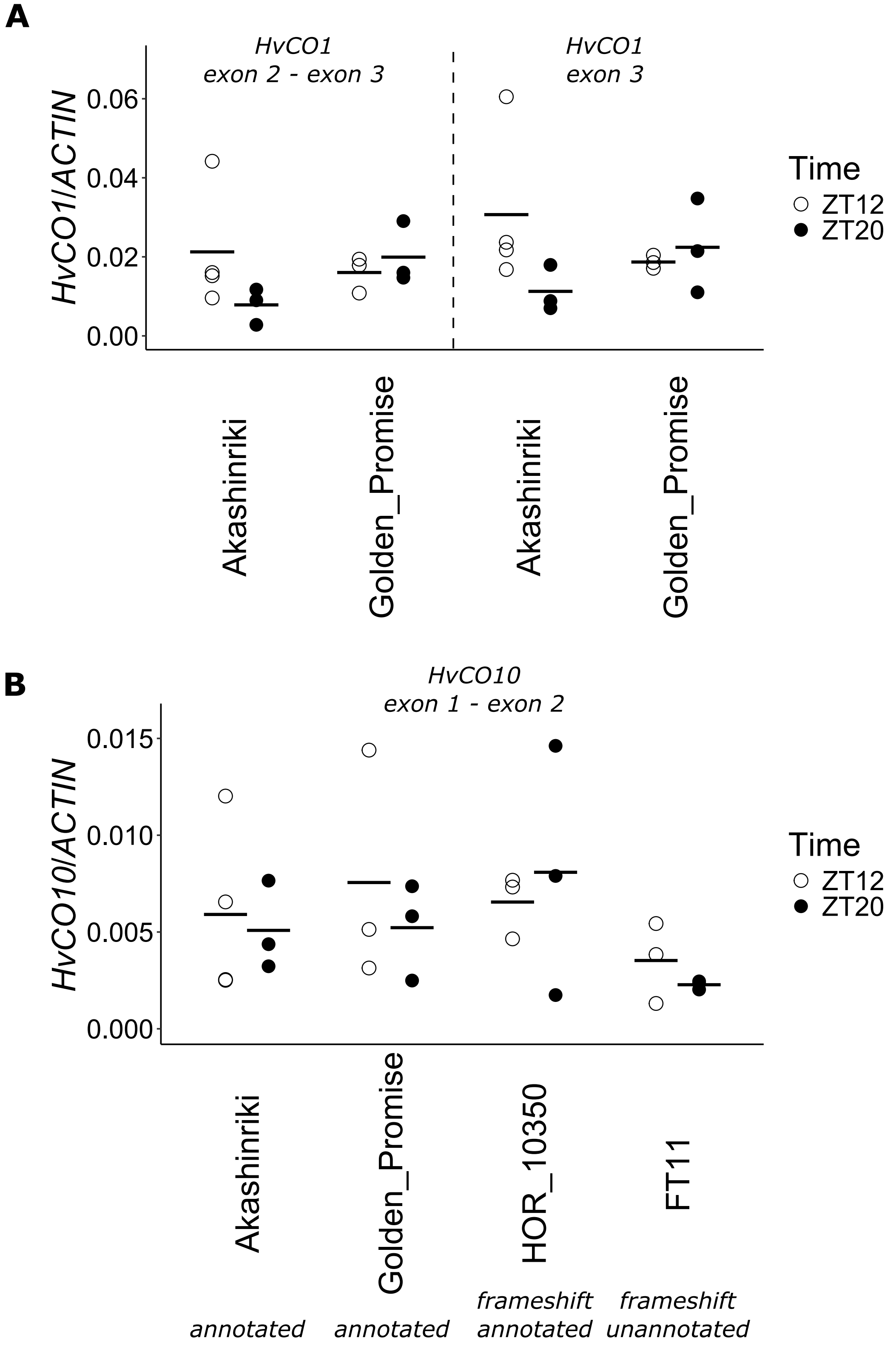

Figure S2. Expression of *HvCO1* and *HvCO10* across genotypic variants. Transcript levels of *HvCO1* (A) and *HvCO10* (B) in selected genotypes. Leaf samples were harvested at ZT12 and ZT20. Expression levels were normalized to *ACTIN*. Horizontal bars represent mean values from biological replicates (n = 3-4).

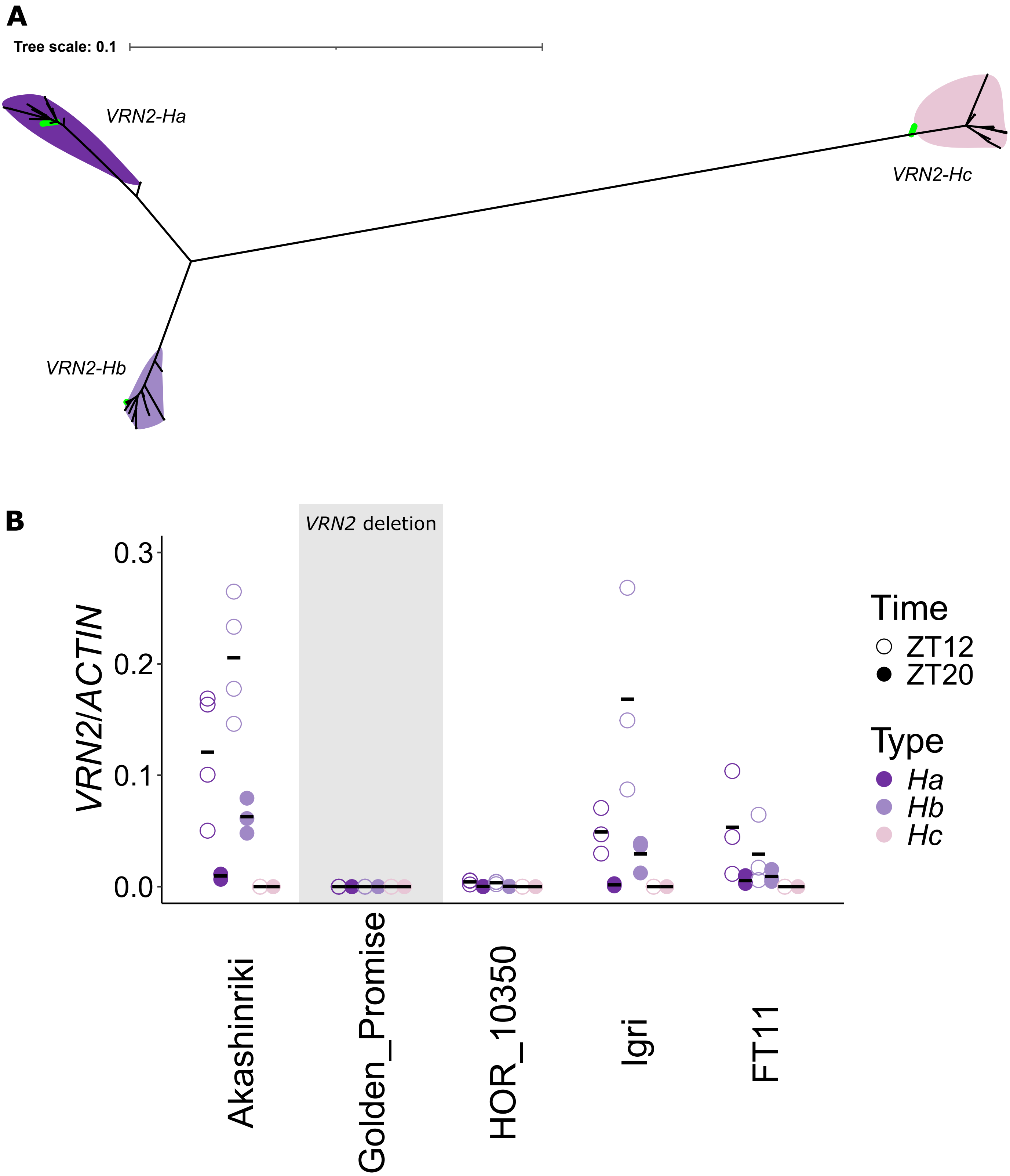

Figure S3. Classification and expression of *VRN2*. (A) Phylogeny of *VRN2* across barley pangenome genotypes. The phylogenetic tree was constructed based on coding sequences using the maximum likelihood IQ-TREE HKY+F+G4 model. *VRN2* copies are classified based on reference sequences (Yan et al., 2004b; Dubcovsky et al., 2005), which are highlighted with neon green branches. (B) Transcript levels of *VRN2* copies in selected genotypes. Leaf samples were harvested at ZT12 and ZT20. Expression levels were normalized to *ACTIN*. Horizontal bars represent mean values from biological replicates (n = 3-4). Golden Promise, shaded in grey, has deletions of all *VRN2* copies.

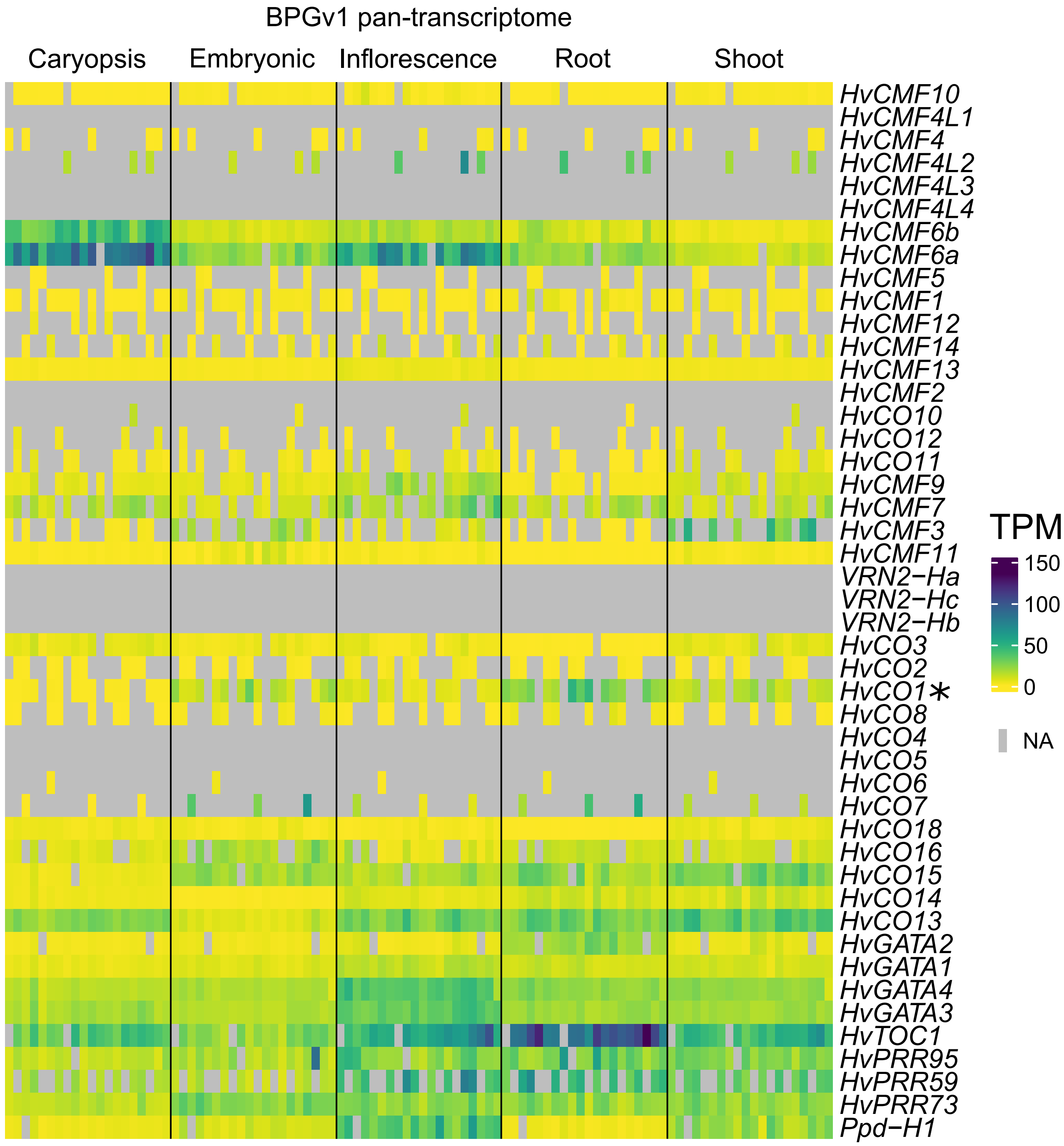

Figure S4. Expression variability of CCT genes across 20 barley genotypes. The heatmap illustrates the average transcript levels (n = 2-3), quantified in transcripts per million (TPM, Guo et al., 2025) for all CCT genes across five tissues. For each tissue, data from 20 genotypes are displayed in the following order (left to right): Akashinriki, Barke, Chi Ba Damai, Du Li Huang, FT11, Golden Promise, Hockett, HOR 10350, HOR 13821, HOR 13942, HOR 21599, HOR 3081, HOR 3365, HOR 7552, HOR 8148, HOR 9043, Igri, Morex, OUN333, and RGT Planet. The gene order is determined by the phylogenetic tree (Figure 3A). *HvCO1* transcripts, indicated by an asterisk, were aligned with two additional neighboring genes (Figure S1A).

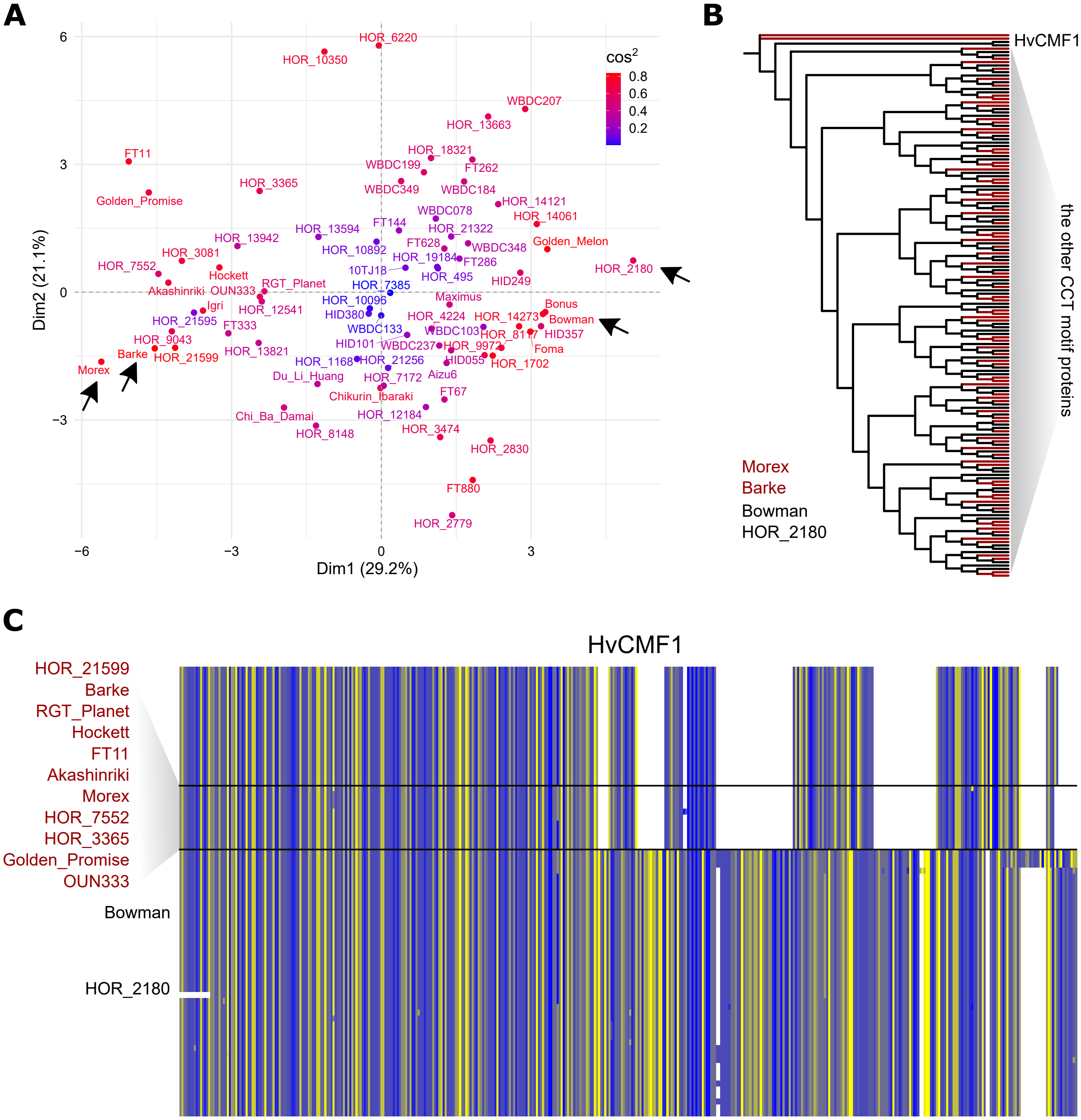

Figure S5. HvCMF1 variation in the barley pangenome. (A) Principal component analysis (PCA) of CCT motif protein variation across the barley pangenome, excluding VRN2. Cosine squared (cos^2) values reflect the contribution of each genotype to the principal components. Arrows highlight selected contrasting genotypes: Morex and Barke versus HOR 2180 and Bowman. (B) Phylogeny of CCT motif proteins, excluding VRN2, in selected genotypes. The phylogenetic tree was constructed using the maximum likelihood IQ-TREE JTT+F+I+G4 model, with branches colored according to genotype groups, emphasizing HvCMF1 as the most divergent protein between groups. (C) Two major HvCMF1 variants identified in the barley pangenome, potentially explaining the genotypic separation observed in (A). Amino acids are color-coded according to the 'Strand' scheme in the sequence alignment.
